# Characterization of Chronic Hepatitis E Virus Infection in Immunocompetent Rabbits

**DOI:** 10.3390/v14061252

**Published:** 2022-06-09

**Authors:** Chunnan Liang, Chenyan Zhao, Tianlong Liu, Bo Liu, Zhiguo Liu, Huili Huang, Wei Liu, Minghai Zhao, Nan Xu, Qiong Lu, Jianhui Nie, Li Zhang, Weijin Huang, Ruiping She, Youchun Wang

**Affiliations:** 1 National Rodent Laboratory Animal Resources Center, Institute for Laboratory Animal Resources, National Institutes for Food and Drug Control (NIFDC), Beijing 102629, China; chunnan_liang@nifdc.org.cn (C.L.); nifdcliuzhiguo@163.com (Z.L.); huanghuili1001@126.com (H.H.); liuwei@nifdc.org.cn (W.L.); zhaominghai@nifdc.org.cn (M.Z.); 2 Division of HIV/AIDS and Sex-Transmitted Virus Vaccines, Institute for Biological Product Control, National Institutes for Food and Drug Control (NIFDC), Beijing 102629, China; zhaochenyan@nifdc.org.cn (C.Z.); xunan@nifdc.org.cn (N.X.); joanlulu@163.com (Q.L.); niejianhui@nifdc.org.cn (J.N.); zhangli@nifdc.org.cn (L.Z.); 3 Laboratory of Animal Pathology and Public Health, Key Laboratory of Zoonosis of Ministry of Agriculture, College of Veterinary Medicine, China Agricultural University, Beijing 100193, China; liutianlong@cau.edu.cn (T.L.); liubo19900409@163.com (B.L.)

**Keywords:** hepatitis E virus, chronic hepatitis E, rabbit model

## Abstract

Chronic hepatitis E virus (HEV) infection is frequently reported in immunocompromised patients, but has also been increasingly reported in non-immunocompromised individuals. We characterized the course of chronic HEV infection in immunocompetent rabbits. In two independent experiments, 40 specific-pathogen-free rabbits were infected with a rabbit HEV genotype 3 strain in serial diluted titers (10^8^ to 10^4^ copies/mL). Serum and fecal samples were collected weekly and were tested for HEV RNA, antigen, anti-HEV and liver enzymes. Rabbits that spontaneously cleared the infection before 10 weeks post-inoculation (wpi) were kept to the end of the study as recovery control. Liver tissues were collected from HEV-infected rabbits at 5, 10 and 26 wpi for histopathological analysis. Nineteen rabbits (47.5%) developed chronic HEV infection with persistent viraemia and fecal HEV shedding for >6 months. Seroconversion to anti-HEV was observed in 84.2% (16/19) of the chronically infected rabbits. Serum levels of aminotransferase were persistently elevated in most of the rabbits. Characterizations of chronic HEV infection in immunocompetent settings could be recapitulated in rabbits, which can serve as a valuable tool for future studies on pathogenesis.

## 1. Introduction

Hepatitis E virus (HEV) is a positive-sense, single-stranded RNA virus and one of the leading causes of acute viral hepatitis worldwide. There are more than 20 million HEV infections globally per year, leading to up to 70,000 deaths [1]. At least four human-pathogenic HEV genotypes falling under the genus *Orthohepeviridae* have been described (HEV1-4) [2]. HEV-1 and HEV-2 strictly confine to humans and are prevalent in developing countries causing waterborne outbreaks via the fecal-oral route. By contrast, HEV-3 and HEV-4 are zoonotic pathogens and the main animal reservoirs are pigs, wild boars and rabbits. These HEV genotypes are transmitted through contaminated animal products in developed countries and in Asia, such as China [1].

HEV infection is generally acute and self-limited. However, solid-organ transplantation recipients and HIV/AIDS patients may progress to a chronic hepatitis E with the risk for the rapid development of liver fibrosis and even cirrhosis [3,4,5]. Moreover, chronic HEV infection cases have also been found in non-immunocompromised individuals [6] and eligible blood donors [7,8], which arouses increasing attention.

Over the past decade, global interest in chronic hepatitis E has increased. Recent studies have developed several interesting immunocompromised animal models that can mimic human chronic HEV infection, including human liver chimeric mice [9,10,11], pigs [12], cynomolgus macaques [13], and rabbits [14]. Chronic HEV infection in immunocompetent animals has been reported in ferrets [15], rhesus macaques [16] and rabbits [17,18]. However, the number of animals used in these studies was relatively limited.

Rabbits are the natural host of HEV-3 and are considered the main reservoir of HEV second to pigs [19]. Both acute and chronic rabbit HEV infection in humans have been reported in recent years [20,21]. We and other research groups previously demonstrated that, in a small scale, experimental HEV-3ra infection in some immunocompetent rabbits can persist for six months [17,18]. Therefore, the rabbit model is probably suitable for monitoring the long-term dynamics of human chronic HEV infection under immunocompetent settings, and a large scale of experiment is warranted. In this study, we report the successful establishment of a repeatable immunocompetent rabbit model for human chronic HEV infection with persistence of infections over six months. Moreover, the magnitude and duration of HEV RNA, antigen (Ag) and antibodies, and the dynamics of liver histopathology were determined.

## 2. Materials and Methods

### 2.1. Ethics Statement

The animal protocol was approved by the Committee of Laboratory Animal Welfare and Ethics of the National Institutes for Food and Drug Control, China (No. 2018B006), and the experiment adheres to the ARRIVE guidelines.

### 2.2. Rabbits, Inoculation and Sample Collection

To increase the repeatability and reliability of our model, we performed two independent experiments (T1 and T2) at different time points. The information on the inocula and groups of infected rabbits is summarized in Table 1. Forty specific-pathogen-free (SPF) rabbits were provided from the National Institutes for Food and Drug Control, China. Prior to HEV inoculation, all animals were tested for alanine transaminase (ALT) and aspartate aminotransferase (AST) to establish the baseline, and were confirmed negative for anti-HEV antibody and Ag by commercial enzyme-linked immunosorbent assay (ELISA) kits (Wantai, Beijing, China), and negative for HEV RNA in fecal/serum by real-time PCR using a One Step Real Time kit (Jinhao, Beijing, China). All experiments were carried out under the manufacturers’ instructions and detailed methods were described in a previous study [22].

The original HEV-3ra inoculum (GenBank accession number: FJ906895) was obtained and prepared from serum samples of a farmed rabbit in a previous epidemiological study [23]. The virus was adapted in a SPF rabbit and was then used as the inoculum in the present study. All rabbits were intravenously inoculated with 3 mL inocula. Serum and feces specimens were collected weekly. Selected rabbits were euthanized at 5-, 10- and 26-weeks post-inoculation (wpi) and liver tissues were collected (Table 1).

### 2.3. Serum Biochemistry Analysis of Rabbits

The clinical biochemistry parameters of the rabbit sera were measured, including blood urea nitrogen (BUN), creatinine (CRE), ALT, AST, γ-glutamyl transferase (GGT), lactate dehydrogenase (LDH) and creatine kinase (CK) using Hitachi 7180 (Hitachi Ltd., Tokyo, Japan).

### 2.4. Histopathology of Liver Tissues

Liver tissues collected from rabbits were fixed in 10% neutral buffered formalin and embedded in paraffin. Specimens were cut into 5 μm serial sections. Slides were stained with hematoxylin-eosin and Masson’s trichrome staining (M & T), and subjected to pathological microscopic examination. Slides were visualized using an inverted fluorescence microscope (Eclipse TI-SR; Nikon, Tokyo, Japan). Detailed methods were described in previous studies [24].

### 2.5. Immunohistochemistry Analysis

Liver tissues collected from rabbits were fixed in 10% neutral buffered formalin and embedded in paraffin. Specimens were cut into 5 μm serial sections. HEV ORF2 proteins was detected in formalin-fixed, paraffin-embedded needle aspirates of liver by immunobiochemistry analysis using a mouse anti-HEV ORF2 monoclonal antibody (AbM59510-9-PU, Beijing Protein Innovation, Beijing, China).

## 3. Results

### 3.1. Successful Establishment of Repeatable Chronic HEV Infection in Immunocompetent Rabbits

Chronic HEV infection has been defined as a persistence of HEV viraemia and/or fecal virus shedding for three months, and several studies adopted this definition as the primary criteria for chronic HEV infection animal model establishment, such as the pig model and the human liver chimeric mice model. However, in clinical studies, most of the chronically infected patients showed a >six months infection [1]. Therefore, in our study, rabbits that showed a persistence of HEV replication for at least 26 weeks were considered chronically infected. Rabbits that cleared HEV infection before 10 wpi were considered acutely infected, and such rabbits were raised along with the chronically infected rabbits till the study ended at 26 wpi as recovery control. One rabbit died at 3 wpi and another at 24 wpi, respectively, due to accident.

**Figure 1:**
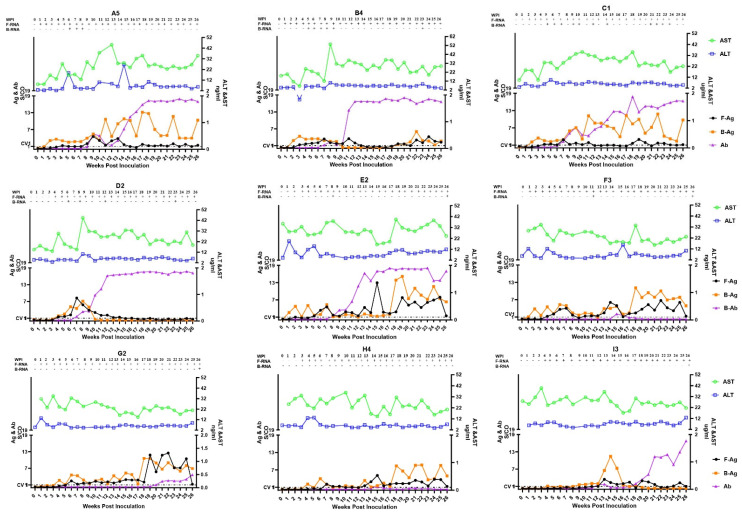
Dynamic of HEV markers and liver function tests in rabbits with chronic HEV infection. (**A5**,**B4**,**C1**,**D2**,**E2**,**F3**,**G2**,**H4**,**I3**) are the number of each representative rabbit. Detection of HEV RNA, HEV antigen, liver function tests, and anti-HEV antibody detection in nine representative chronically infected rabbits, one rabbit per group. Serum and fecal samples were collected weekly for immediate analysis. “-” for negative and “+” for positive of HEV RNA detection by RT-qPCR. Ab, antibody; Ag, antigen; ALT, alanine aminotransferase; AST, aspartate aminotransferase; B, blood; F, feces; HEV, hepatitis E virus; WPI, weeks post-inoculation.

Chronic HEV infection in SPF rabbits was successfully established after intravenously (i.v.) injection of serial diluted HEV inocula (Figure 1). Indeed, 55.0% (11/20) and 40.0% (8/20) of the rabbits in T1 and T2, respectively, developed chronic HEV infection defined as at least 26 weeks of viraemia/fecal virus shedding. In the two independent experiments, T1 and T2, some rabbits in each group presented with persistent fecal shedding of HEV, regardless of the titers of the initial inoculum. Rabbits inoculated with 10^6^ copies/mL and higher titers inocula showed fecal HEV shedding as soon as 1 wpi. Viraemia was detected intermittently positive in all groups, but was undetectable in rabbits of group H and I in T2. The chronicity rates from high to low dose groups were 58.3% (7/12), 62.5% (5/8), 37.5% (3/8), 37.5% (3/8) and 25% (1/4), respectively.

Seroconversion to anti-HEV was observed in most of the rabbits (Figure 1). In rabbits showing acute HEV infection in both experiments, 88.9% (8/9) had detectable anti-HEV antibodies and the S/CO ratio peaked before 10 wpi. However, in chronically infected rabbits, the anti-HEV antibodies level had a delayed increasing trend and 63.2% (12/19) peaked at around 20 wpi. Similar to previous studies of HEV-infected immunocompromised patients and eligible blood donors [7,8], there were five chronically infected rabbits (3/19, 15.8%) that did not seroconvert to anti-HEV throughout the whole study period. HEV ORF2 Ag detection has recently been proven as a promising new index for HEV infection diagnosis and disease development [1]. Therefore, we monitored the HEV-Ag level by using a commercial ELISA kit (Wantai, Beijing) in serum and feces in all rabbits during the whole study (Figure 1). The results were consistent with HEV RNA detection. All chronically infected rabbits but one in group D had detectable HEV-Ag till 26 wpi. Interestingly, in most of the rabbits, serum HEV-Ag level is higher than that of the feces at most of the times. Rabbits that did not develop chronic HEV infection were almost all negative for serum and fecal HEV-Ag, or showed a transient elevation of HEV-Ag at the early phase of infection. However, HEV-Ag in chronically infected rabbits’ samples could be detected as early as 1 wpi and remain positive during the study period.

**Figure 2:**
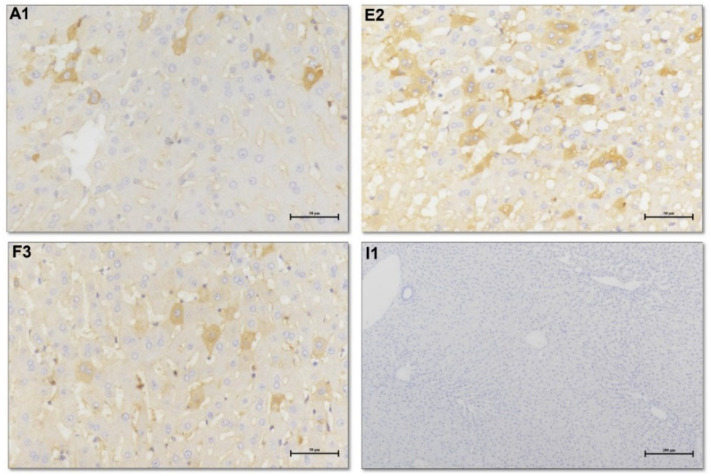
Immunohistochemistry analysis of HEV ORF2 proteins in rabbits’ liver tissues at 26 wpi. (**A1**,**E2**,**F3**) represents the liver tissues of three individual rabbits with chronic infection of HEV. (**I1**) is the liver tissue of the control rabbit in which the HEV infection was cleared within 8 weeks, but was kept in the study until 26 wpi.

We also performed immunohistochemistry (IHC) analysis of HEV ORF2 proteins on three rabbits’ liver tissues at 26 wpi. The results demonstrated positive signals of HEV ORF2 proteins in the chronically-infected rabbits’ liver tissues indicating HEV replication at 26 wpi (Figure 2). One rabbit cleared the HEV infection within 8 weeks, but was kept in the experiment until 26 wpi to be used as negative control (or recovery control), and no positive staining was found in its liver tissue (Figure 2).

**Figure 3:**
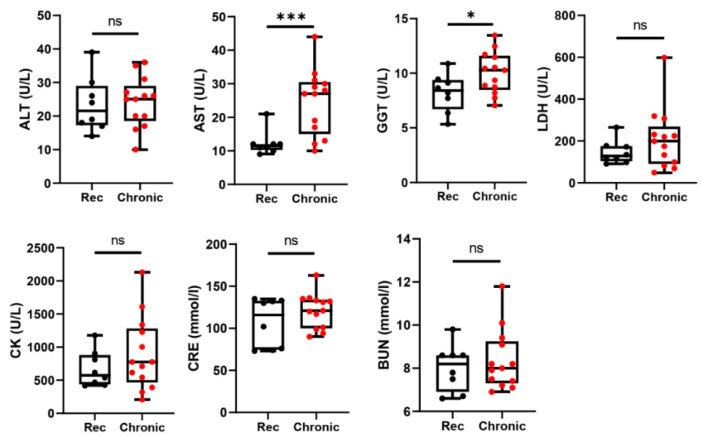
Serum biochemistry in euthanized rabbits at the end of the study**.** At 26 wpi, rabbits with chronic HEV infection (designated as “Chronic”) and rabbits who cleared HEV infection before 10 wpi (designated as “Rec”, serve as recovery control) were euthanized for extended analysis of serum biochemistry. ALT, alanine transaminase; AST, aspartate aminotransferase; BUN, blood urea nitrogen; CK, creatine kinase; CRE, creatinine; GGT, γ-glutamyl transferase; LDH, lactate dehydrogenase; ns, not significant. * *p* < 0.05, *** *p* < 0.001.

Serum levels of liver enzymes ALT and AST were measured during the HEV infection (Figure 1). The majority of rabbits presented with mild and persistent liver function abnormalities. ALT level was generally normal or only very slightly increased during the acute phase (before 10 wpi). However, we observed a persistent elevation of serum AST levels in some rabbits. At 26 wpi, we euthanized rabbits from the recovery control group and chronically infected group, and collected adequate amounts of serum for biomedical analysis. Serum levels of AST and GGT of chronically infected rabbits were significantly higher than that of the recovery rabbits (Figure 3). The average levels of blood urea nitrogen and serum CRE were higher in the chronically infected rabbits than in recovery rabbits, but there was no statistically significant difference.

### 3.2. Course of Liver Histopathology in the Rabbit Model of Chronic HEV Infection

**Figure 4:**
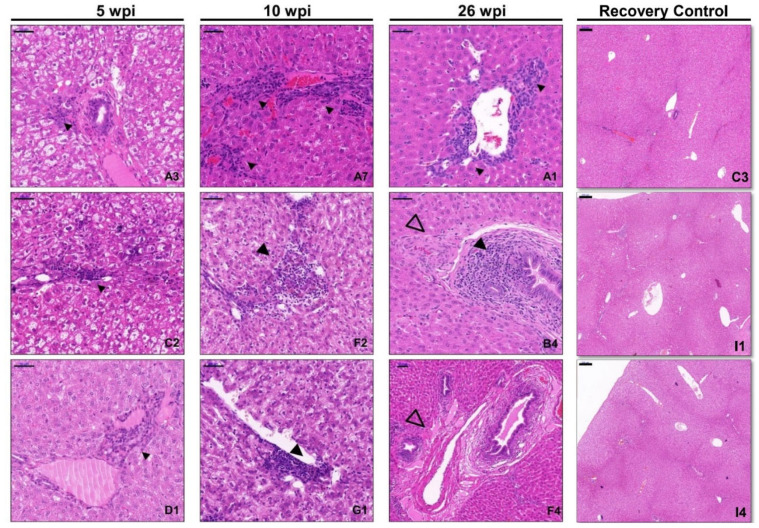
Dynamic of histopathology of liver tissue in HEV-infected rabbits. The number of each rabbit was indicated on the lower right of each image. Liver tissues were collected from HEV-infected rabbits at 5 (**A3**,**C2**,**D1**),10(**A7**,**F2**,**G1**), 26 wpi (**A1**,**B4**,**F4**) and from the recovery group (**C3**,**I1**,**I4**). Inflammatory cell infiltrates and hepatocyte necrosis were found (arrowhead) in all tissues and progressed as infection proceeded. At 26 wpi, chronic inflammatory cells infiltrate the portal area, blood vessel walls thickening associated with fibrosis (empty arrowhead) and local hyaline degeneration was found. No obvious lesion was found in the liver tissues of the recovery group. HEV, hepatitis E virus; wpi, weeks post-inoculation. Bars in the images of 5 wpi, 10 wpi and 26 wpi indicate 50 μm. Bars in the images of recovery group indicate 200 μm.

In T1 and T2, rabbits that tested positive for fecal HEV shedding or viraemia at 5, 10 and 26 wpi, respectively, were euthanized for necropsy (Figure 4). No gross lesions were observed in livers from rabbits at 5 wpi, only scattered infiltrate of inflammatory cells observed in the portal area. Lesions were more severe at 10 wpi as lymphocytes distributed focal or scattered in hepatic lobule and the inflammatory cells recruited along blood vessel walls. Bridging necrosis or piecemeal necrosis, mild congestion and cellular swelling was also observed. However, rabbits infected with lower viral titers in group D and I showed no obvious lesions.

Little is known about the long-term progression of chronic HEV infection-induced liver pathology in large-scale animal model studies. We obtained the liver tissues from chronically infected rabbits at 26 wpi for detailed histological analyses (Figure 4). H&E staining showed mild piecemeal necrosis and moderate inflammation around the portal vein, demonstrating chronic hepatitis. Fibrous septum with some ductular proliferation and slight interface hepatitis was also observed. These results showed that the rabbit model displayed the typical pathology characteristics observed during the course of chronic HEV infection, which indicated that these rabbits exhibited a chronic hepatitis pattern similar to the chronic hepatitis E patients. Rabbits that cleared HEV infection within 8 weeks were kept to the end of the experiment and were used as the recovery control. No obvious lesion was found in the liver tissues of the recovery group (Figure 4).

## 4. Discussion

In recent years, chronic hepatitis E has gained more attention worldwide and has been recognized as an emerging significant clinical challenge not only confined to immunocompromised patients but also to immunocompetent individuals [6,7,8]. The lack of a desirable small animal model for chronic HEV infection hinders our understanding of the pathogenesis and the development of therapeutics measures. In the present study, by infecting immunocompetent rabbits with a strain of HEV3, we established a model that could produce robust chronicity. The present rabbit model for chronic HEV infection can be successfully repeated and reproduced, making the model more reliable in the future application.

This study provided the opportunity to examine the long-term viral kinetics of HEV during the chronic infection. Fecal virus shedding in rabbits is the most sensitive and stable marker of HEV infection and can appear as early as 1 wpi. All rabbits that developed chronic hepatitis E had persistent fecal virus shedding till the end of the study at 26 wpi. Serum viral RNA of the infected rabbits persisted for a much shorter period of time when compared to fecal virus shedding. This finding is in line with previous studies of pig and rabbit models of chronic HEV infection [12,14,18] as fecal HEV shedding is more stable and persistent than viraemia. The potential mechanism of this phenomenon warrants future investigation. Recent clinical reports demonstrated that persistence of HEV RNA in the feces at the end of ribavirin therapy in patients with undetectable HEV RNA in blood was associated with a higher risk of HEV infection relapse [25,26]. Therefore, HEV viraemia alone may not be a sufficient indicator of chronic HEV infection. HEV-Ag is a novel marker of HEV infection that has been studied in many clinical studies [7,27,28]. We also investigated the kinetics of HEV-Ag in the serum and fecal samples of the rabbits as the long-term kinetics of HEV Ag detection during HEV infection is not known. It is interesting that most of the rabbits that developed chronic hepatitis E present with persistent antigenemia at the early phase of the infection. However, those rabbits that spontaneously cleared the infection within 8 wpi showed almost no HEV antigenemia or fecal antigen shedding during the whole experiment. This observation agrees well with the result of a recent clinical study conducted in solid-organ transplant patients that higher serum HEV-Ag concentration at the acute phase of HEV infection may predict progression of chronic infection [27]. A dramatic increase of HEV-Ag level in serum and feces of most of the chronically infected rabbits was observed after 13 wpi, which suggested accumulation of a secreted form HEV capsid proteins and active HEV replication at the chronic phase of infection. Although the fecal HEV shedding in rabbits is more stable than viraemia, the level of serum antigen is higher than in feces even in HEV RNA-negative samples. Similar findings are also observed in humanized mice [29]. Our results indicated that the detected antigen likely corresponds to noninfectious ORF2 proteins, as previous studies demonstrated that noninfectious ORF2 proteins are the major antigens in cell culture supernatant and patient sera [30,31].

Although the liver histopathology has been reported in immunocompromised chronic HEV patients, little is known about the liver histopathology in immunocompetent patients with chronic HEV. The dynamics of liver histopathology was studied in the present immunocompetent rabbit model. At the acute phase, inflammation and lymphocyte infiltration is the major observation with spotty necrosis. At the chronic phase, liver tissues of rabbits showed features of chronic viral hepatitis. These findings are similar to chronic hepatitis E patients [32,33]. We used the rabbits recovered from acute HEV infection as a control in order to further exclude confounding factors during infection, and no obvious lesion was observed at 26 wpi. The chronic lesions in the liver were also supported by serum biochemical analysis of liver enzymes. The level of AST showed persistent mild-to-moderate elevation in most of the chronically infected rabbits during the whole study period, indicating ongoing liver injury. GGT level was significant higher in rabbits with chronic hepatitis E than that of the recovery rabbits at 26 wpi. One of the limitations of this study is that no negative control group was set during the study. Although a progression of liver pathology was found, the lesions of the liver tissues observed at 5 and 10 wpi should be interpreted cautiously.

In summary, the immunocompetent rabbit model provides a repeatable and promising tool for future study of the pathogenesis and underlying mechanism for why chronic HEV can also occur in immunocompetent individuals.

## Figures and Tables

**Table 1:** Design of the experimental HEV infection in SPF rabbits.

Experiment	Group	Infectious Dose (Copies/mL)	Necropsy at 5 Wpi	Necropsy at 10 Wpi	Necropsy at 26 Wpi	Chronic Infection
T1	A (n = 8)	10^8^	1	1	2	5
	B (n = 4)	10^7^	1	0	2	2
	C (n = 4)	10^6^	1	0	2	2
	D (n = 4)	10^5^	1	0	2	2
T2	E (n = 4)	10^8^	0	1	3	2
	F (n = 4)	10^7^	0	1	3	3
	G (n = 4)	10^6^	0	1	2	1
	H (n = 4)	10^5^	0	1	3	1
	I (n = 4)	10^4^	0	1	3	1

HEV, hepatitis E virus; SPF, specific-pathogen free; wpi, weeks post-inoculation.

## Data Availability

Not applicable.
